# Study on a Luminol-based Electrochemiluminescent Sensor for Label-Free DNA Sensing

**DOI:** 10.3390/s101009481

**Published:** 2010-10-21

**Authors:** Hai-Hong Chu, Ji-Lin Yan, Yi-Feng Tu

**Affiliations:** Institute of Analytical Chemistry, Department of Chemistry, Soochow University,Dushu Lake Higher Education Town, China-Singapore Suzhou Industrial Park, Jiangsu, 215123, China; E-Mails: chuhaihong@suda.edu.cn (H.-H.C.); yanjl@suda.edu.cn (J.-L.Y.)

**Keywords:** DNA, label-free sensing, electrochemiluminescence, nano-prefunctional sensor, quenching effect

## Abstract

Automatic, inexpensive, simple and sensitive methods for DNA sensing and quantification are highly desirable for biomedical research. The rapid development of both the fundamentals and applications of electrochemiluminescence (ECL) over the past years has demonstrated its potential for analytical and bio-analytical chemistry. This paper reports the quenching effect of DNA on the ECL of luminol and the further development of a DNA sensing device. With the pre-functionalization by a composite of carbon nano-tubes (CNTs) and Au nanoparticles (AuNPs), the sensor provides a novel and valuable label-free approach for DNA sensing. Here the ECL intensity was remarkably decreased when more than 1.0 × 10^−12^ molar of DNA were adsorbed on the sensor. Linearity of the DNA amount with the reciprocal of ECL intensity was observed. A saturated sensor caused a 92.8% quenching effect. The research also proposes the mechanism for the quenching effect which could be attributed to the interaction between luminol and DNA and the elimination of reactive oxygen species (ROSs) by DNA.

## 1. Introduction

Rapid detection, quantification and sequencing of deoxyribonucleic acid (DNA) are important tasks in the fields of biology, drug discovery, medical diagnostics, agriculture, as well as environmental science *etc*. In the past decades, abundant research has focused on this topic [1]. Automatic, inexpensive, simple and sensitive methods such as spectrophotometry, electrochemical method, fluorescence, chemiluminescence and some others were proposed [2–4]. Electrochemiluminescence (ECL) could be described as a synchronous or succedent chemiluminescence (CL) as the result of an electrochemical reaction [5–7]. As an analytical technique, ECL possesses favorable advantages [8,9]. It is more repeatable due to the fact the light-emitting process can be accurately controlled for synchronization and dimensional orientation with the electrochemical reaction on electrode surface. Additonally the ECL detection was separated from the electrochemical excitation, which is beneficial for enhancement of signal/noise ratio and therefore the sensitivity. ECL can be controlled by manipulating the applied potential and/or other electric parameters to realize selective detection. In the ECL procedure, more than one of the reactive species is electrochemically produced *in situ*, therefore avoiding the problems associated with the use of some reactive and unstable chemicals. In addition, the ECL instrument is often simpler than many electrochemical or fluorescence instruments. One of the most extensively used ECL reagents is luminol due to its high efficiency in aqueous solution under appropriate conditions [10].

Recently, more interest has been devoted to DNA sensing devices such as piezoelectric, optical or electrochemical sensors [11]. The typical design of a DNA sensor involves the appropriate immobilization of the DNA molecules or introduction of an effective indicator or so called labeled probe. In this field, nano-material modified electrodes are inherently ideal because of their enormous specific surface, which is highly susceptible to heterogeneous redox reactions with the surrounding environments. It has received considerable attentions due to the fascinating electrochemical, electrocatalytical and biocompatible properties. Typically, carbon nano-tubes (CNTs) have attracted increasing attention [12,13]. Their promising applications are based on the promotional ability of electron-transfer with target molecules in some electrochemical reactions. The CNT-modified electrode showed better performance due to the size effect, electronic structure and exterior topological characteristics. Another important nano-material, Au nanoparticles (AuNPs), is usually used to align and electrically contact biomolecules with electrode supports. It may also be used to enhance the charge transfer, thereby to accelerate bio-electrocatalytic processes [14,15]. As reported, it was thought that AuNPs could facilitate the hybridization in a novel approach to telomerase activity detection with ECL [16].

For DNA sensing, Will *et al*. studied the fluorescence quenching reaction of luminol with short DNA fragments employing steady-state and time correlated single photon counting technique [17]. It was proposed that the phosphate backbone present in DNA was responsible for the interaction with luminol. Lee *et al*. have developed a high sensitive Ru(bpy)_3_^2+^ based ECL method using daunorubicin (DNR) and doxorubicin (DOX) as coreductants in aqueous solution for various pathogenic DNA samples [18]. They studied the characteristics of ECL reaction with some intercalators to apply DNA hybridization detection, too. Tomás Pérez-Ruiz, *et al*. proposed an automatic method of coupling a photochemical reaction and a CL reaction in a flow-injection system to obtain a reliable method for the determination of DNA [19]. An ultrasensitive DNA hybridization detection method based on ECL using polystyrene microspheres/beads (PSB) as the carrier of the ECL labels was reported [20]. A bio bar code assay based on oligonucleotide-modified AuNPs provided a PCR-free method for quantitative detection of nucleic acid targets [21]. A review presented a general picture of the latest progress and developments related to novel nanomaterials for ECL-based biosensors [22]. It briefly covered the basic mechanisms of ECL detection, and the recent developments in fabrication of solid-state ECL sensors using nanomaterials.

Herein, we will report our research on a new designed label-free approach for DNA sensing using the ECL of luminol as readout signal on a CNTs/AuNPs composite pre-functionalized electrode. The results indicated that the ECL response was correlated with the presence of dsDNA. Based on the research, the possible quenching mechanism of dsDNA for ECL of luminol has also been discussed. It might result from the interaction of luminol with dsDNA, and the elimination of reactive oxygen species (ROSs) by dsDNA. Here the ROSs have already been proven as efficient ECL intensifiers of luminol [23,24]. Comparative methods as electrochemistry, UV-Vis and fluorescence spectra have supported the ECL discussion [25–27].

## 2. Experimental Section

### 2.1. Apparatus

A 5 mL tubular bottle is used as the ECL cell. The bottle is completely shielded with a silver-mirror film, leaving only a small window at the middle of its bottom to allow the transmission of light. A glassy carbon (GC) electrode is used as the working electrode. A platinum wire and an AgCl coated silver wire act as the auxiliary and reference electrodes, respectively. The transparent window of the ECL cell is mounted above the center of a photoelectric multiplier (PMT), which works to detect the ECL intensity, and both are shielded in a black box. The PMT is powered by a negative high voltage supplier and the photocurrent recorded by the computer through an A/D interface. A BAS-100A Electrochemical Analyzer (Bioanalysis System Inc., USA) is used as a potentiostat to exert the electrolytic potential with a self-designed digital-pulse-generator as the outer pulse source. Fluorescence measurements are carried out on an F-4500 Fluorescence Spectrophoto-meter (Hitachi, Japan). UV-Vis spectra are obtained from a TU-1810 Spectrophotometer (Beijing Purkinje General Instrument Co., Ltd.). The microscopical images are taken on H-600(II) Transmission Electron Microscope (Hitachi, Japan). A CHI-660a Electrochemical Workstation (Chenhua Instruments Co., Ltd., Shanghai, P.R. China) is used in the electrochemical studies.

### 2.2. Chemicals

Luminol (AG) was purchased from Fluka and used without further purification. Stock solution of 0.01 mol L^−1^ was prepared by dissolving luminol in 0.2 mol L^−1^ NaOH solution and stored in a refrigerator. Calf thymus DNA (ct-DNA, double-stranded with molecular weight of about 10^5^D) was purchased from Sigma. The 1.0 × 10^−6^ mol L^−1^ dsDNA stock solution was prepared by dissolving it in doubly distilled water and kept in the refrigerator. The synthetic oligonucleotide which acted as a model of ssDNA was purchased from Shenggong Bioengineering Co. Ltd. (Shanghai, China). Its base number is 24. The base sequence is 5′-NH_2_-GAG CGG CGC AAC ATT TCA GGT CGA-3′. Its solution was prepared with water and kept in the refrigerator. CNTs were purchased from Shenzhen Bill Technology Developing Co. Ltd. They were sonicated in concentrated nitric acid at 25 °C for about 24 h, then purified through filtration and washed with double-distilled water until the filtrate became neutral then dried in an oven. AuNPs were prepared according to literature [27]. The diameter was about 30 nm, which was measured by TEM. All other reagents were of analytical grade. Doubly distilled water was used throughout the experiments.

### 2.3. Preparation of DNA ECL Sensor

**Figure 1.**
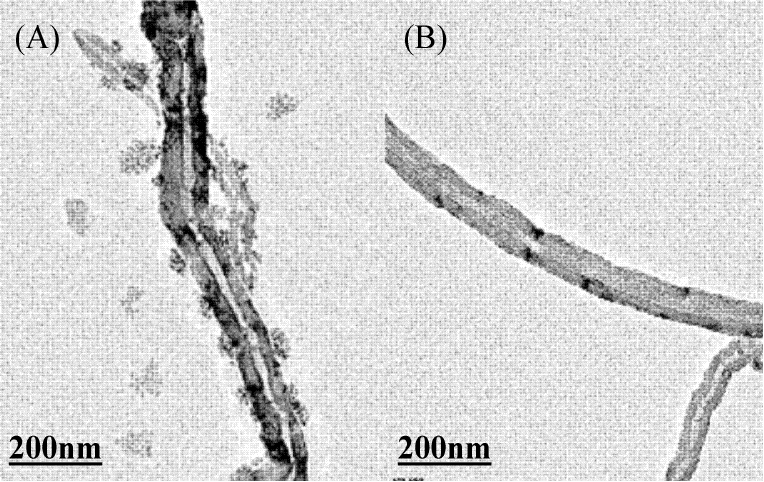
The TEM images of **(A)** CNTs/AuNPs composite and **(B)** CNTs.

A dilute slurry of AuNPs/CNTs composite was obtained by mixing AuNPs and CNTs in a proportion of 3:4 in a test tube, followed by sonication for 30 min. As shown in Figure 1, the TEM images demonstrated a well-proportioned conglutination of AuNPs on CNTs. The working electrode was first polished to a mirror-like plate with 0.05 μm alumina slurry on micro-cloth pads. The cleaned electrode was then dip-coated with the CNTs/AuNPs slurry and dried under an infrared lamp. For loading the target DNAs, the pre-functionalized electrode was soaked into 1mL of dsDNA or ssDNA solution for enough time to ensure the adsorption. To study the effect of loading amount of dsDNA on ECL response, different volume of 1.0 × 10^−6^ mol L^−1^ dsDNA solution was dripped onto the surface of nano-functionalized electrode and kept for immobilization. Finally, the electrodes were rinsed thoroughly with distilled water and kept in the refrigerator at 4 °C before use.

### 2.4. General Procedure

In ECL measurements, the test solutions with appropriate amounts of buffer and luminol were pipetted into the ECL cell. The ECL signal was excited by the electrolytic pulse with 1.5 V of the upper limiting potential, 0 V of the lower limiting potential, 6 s of the period and duty factor of 1:5. Several types of neutral and weak alkaline media such as borate, phosphate, citrate and borate-phosphate buffer solutions were tested for study of the effect on the ECL measurement. The phosphate buffer solution was found to be the best one to produce high quality ECL signal. The pH of medium which influenced the response will be investigated in detail.

To detect the DNA content, 10 μL of DNA contained solution was dripped onto the pre-functionalized electrode, after waited for several minutes, the sensor was applied to detect the ECL intensity in phosphate buffer of pH8.0 which containing 1.0 × 10^−7^ mol L^−1^ of luminol. The DNA content could be calculated from the regression curve of ECL response with DNA content.

The fluorescence spectra of dsDNA, luminol and their mixed solution were taken at 260 nm of exciting wavelength. The effect of dsDNA on UV-Vis spectra of luminol was obtained by adding a certain volume of dsDNA into luminol solution.

## 3. Results and Discussion

### 3.1. The Quenching Effect from DNA on ECL of Luminol in Solution

**Figure 2.**
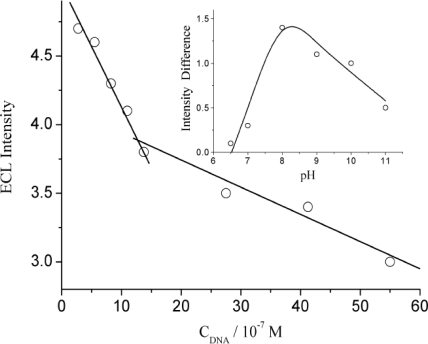
ECL response on bare GC electrode upon DNA content in solution containing 5.0 × 10^−5^ mol L^−1^ luminol. Insert the ECL response on pH of solution.

As illustrated in Figure 2, the dsDNA quenched the ECL of luminol in water, the ECL intensity responded to the concentration of dsDNA negatively in regionalized linearity.

**Figure 3.**
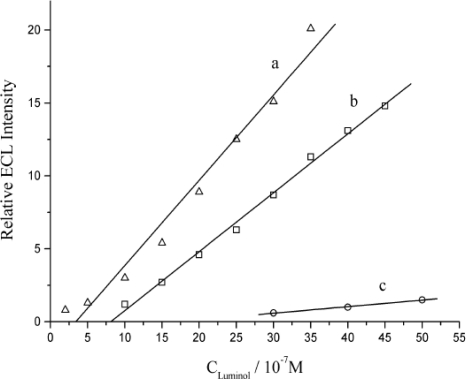
The ECL responses of luminol on **(a)** CNTs/AuNPs/GC electrode, **(b)** ssDNA modified and **(c)** dsDNA modified CNTs/AuNPs/GC electrode.

The quenching effect of dsDNA on ECL of luminol was found to be related to the pH of the media. As shown in the insert of Figure 2, the quenching effect reached the maximum when the solution acidity was around pH 8.0. Therefore, the quenching effect of dsDNA on ECL is partly considered to be attributed to the interaction between these two species. Under low pH, positive charged phosphate backbone of dsDNA and positive charged oxidized intermediate of luminol would obstruct the interaction due to the repulsion force. As the pH gets higher, even though the ECL of luminol is stronger, it didn’t show more distinguished quenching effect by dsDNA. It has been well documented that alkaline denaturalization could happen and dsDNA would split into single-stranded DNA (ssDNA) at high pH, the interaction between luminol and ssDNA was deduced to be weak [28]. The experimental results in Figure 3 can testify the discussion, too.

Figure 3 shows the ECL responses of luminol on different electrodes. It can be seen that all the ECL emission responded to the luminol at those electrodes linearly. It is notable that the ECL response slopes decrease in the order of CNTs/AuNPs/GC electrode, ssDNA modified, and dsDNA modified electrodes, but the difference between nano-functionalized electrode and ssDNA modified electrode is remarkably smaller than with dsDNA modified electrode. Here the dsDNA quenched 92.8% of ECL intensity but ssDNA only 30.7%. The results proved that the quenching ability of ssDNA was relatively weaker than that of dsDNA, so is not difficult to understand the lower quenching effect of dsDNA under high pH.

On the other hand, as it has been reported that ROSs could enhance the ECL of luminol in neutral and weak alkaline solutions [17,23,29,30], after stable ECL signals were obtained at a certain concentration of hydrogen peroxide, the ECL intensity started to decrease toward the background even lower along with the addition of dsDNA. It revealed the elimination of those radical(s) from H_2_O_2_ reduction as hydroxyl radicals (HO^•^) and superoxide anion radicals (O_2_^•–^) by dsDNA hence minimized the enhancing effect on ECL. So the dividually linear ECL response of luminol in different concentration range in Figure 2 could be attributed to those two dominant effects of the different quenching mechanism.

### 3.2. The Electrochemical Behavior of DNA Modified Electrode

**Figure 4.**
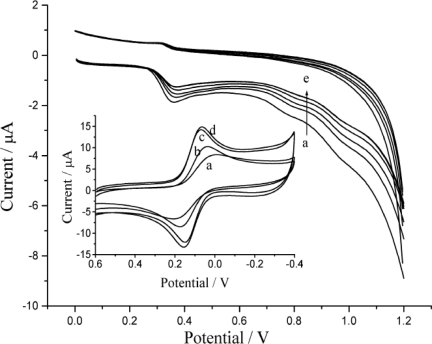
Cyclic voltammograms of 5.0 × 10^−5^ mol L^−1^ luminol in PBS of pH 8.0 with the addition of DNA for **(a)** 0, **(b)** 2.8 × 10^−7^ mol L^−1^, **(c)** 5.5 × 10^−7^ mol L^−1^, **(d)** 8.2 × 10^−7^ mol L^−1^ and **(e)** 1.1 × 10^−6^ mol L^−1^. Insert are cyclic voltammograms on **(a)** bare GC electrode, **(b)** DNA/CNTs/AuNPs/GC electrode, **(c)** CNTs/AuNPs/GC electrode and **(d)** CNTs/GC electrode in buffer solution containing 0.1 mol L^−1^ K_3_[Fe(CN)_6_]. Both at the scan rate of 100 mV/s.

The insert of Figure 4 shows the cyclic voltammograms of probe ion (Fe(CN)_6_^3−^) on different electrodes. Theoretically, Fe(CN)_6_^3−/4−^ exhibits a reversible redox process with about 60 mV of peak-to-peak separation on ideal electrode typically as gold or platinum. But the glassy carbon possess larger resistance than common carbon materials or metal materials, and also the surface of glassy carbon is not so easy to be activated, so it is difficult to obtain the ideal CV property on a simple polished glassy carbon electrode unless to pretreat it with strong oxygenic acid or electro-oxidation. In general, it displays a couple of flat and wide peaks of redox current just as illustrated curve (a). Since nano-particles (NPs) exhibit higher surface ratio than their bulk counterparts [31] the NPs-modified electrochemical interfaces will provide larger electro-active areas and therefore lead to higher response for target molecules. CNT plays a complementary role as a support which serves as a fast electron-transfer center and detects the catalytic oxidation current [32,33]. AuNPs can easily act as enhancing agents for effective acceleration of electron transfer, leading to more rapid current response, so it is not difficult to understand that both the CNTs and CNTs/AuNPs modified electrode achieved much higher peak currents and smaller peak-to-peak separation than a bare GC electrode. These facts strongly indicated that CNTs and AuNPs could promote the redox process of Fe(CN)_6_^3−^. The effect of dsDNA on the modified electrode presented as line b in insert of Figure 4. In direct contrast, both the decreased current response and the poor peak-to-peak separation (135 mV) suggested that the immobilized dsDNA on the electrode would obstruct the probe ions’ redox reaction.

Since the performance is related to the DNA loading, the influence of the amount of DNA on the electrochemical response of luminol was studied. As illustrated in Figure 4, the anodic peak currents of luminol decreased correspondingly to the loading amount of DNA. This phenomenon should be attributed to gradual adsorption of DNA on the electrode surface [34].

### 3.3. The Quenching Efficiency of Immobilized DNA for ECL of Luminol

**Figure 5.**
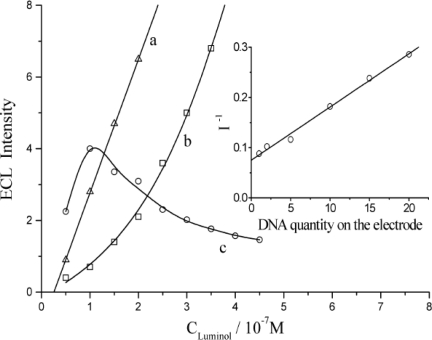
The ECL responses of luminol on **(a)** bare GC electrode and **(b)** dsDNA modified electrode. Curve **(c)** is the multiples of **(a)** to **(b)**. Insert is the regression curve of the reciprocal of ECL intensity (I^−1^) *versus* the adsorptivd dsDNA quantity. Here the X axis presents the volume (μL) of 1.0 × 10^−6^ mol L^−1^ dsDNA solution which was used.

The ECL response of luminol on this DNA ECL sensor was examined and the results are shown in Figure 5. Similar to the previously mentioned phenomenon, ECL intensity decreased with the loading amount of DNA. The ECL signals of luminol obtained at the DNA modified electrode (curve b) showed significant differences from those produced at a bare GC electrode (curve a). The relative quenching effect is variably related with the amount of DNA loaded as presented in curve c, multiplying the ECL intensity on the bare electrode over that of the dsDNA modified electrode. The value reached the maximum at 1.0 × 10^−7^ mol L^−1^ concentration of luminol. This concentration was therefore adopted as the optimal condition for further research. At this concentration of luminol, a linear response was obtained between the reciprocal of ECL intensity (I^−1^) and the loading amount of DNA on electrode surface (see insert of Figure 5) between the loading amount of DNA from 1.0 × 10^−12^ molar to 2.0 × 10^−11^ molar.

The repeatability and stability of the DNA ECL sensor was also investigated. It was found that the RSD equaled to 1.76% with eleven sequential detections and the ECL intensity decreased only about 8% after storage at 4 °C for 30 days. The good long-term stability could be contributed to the CNTs and AuNPs composite matrix, which provided a biocompatible microenvironment.

Some organic molecules and drugs might interact with DNA even damage to DNA, they probably cause the interference for ECL response of this sensor. In our research, as a model, the interference of chloramphenicol (CAP) had been examined. The results revealed that more than 1.0 × 10^−6^ mol/L of CAP would cause the interference for ECL detection of DNA. Considering the procedure of sample preparing such as separation, there mustn’t be so concentrated organic molecules or drugs, they wouldn’t cause obvious interference for this sensor.

### 3.4. Discussions on Quenching Mechanism of DNA on ECL Response

**Figure 6.**
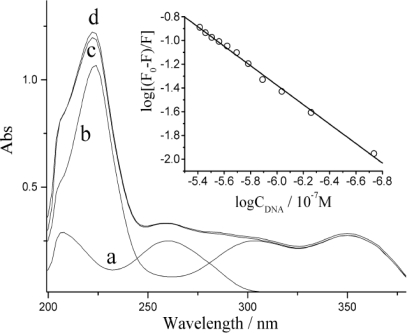
The UV-Vis absorption spectra of (a) 1.1 × 10^−7^ mol L^−1^ dsDNA, (b) 4.0 × 10^−5^ mol L^−1^ luminol, (c) the mixture of a and b and (d) overlapped mathematically of a and b in PBS of pH 8.0. Insert is the Stern-Volmer plot from fluorescence, DNA, C_Luminol_ = 5.0 × 10^−6^ mol L^−1^.

When luminol and DNA solutions were mixed together, the thus-obtained UV-Vis spectrum shows only the mathematical overlap of the characteristic spectral features of two individual solutions (see Figure 6). This may suggest no chemical reaction between DNA and luminol.

An emission band on fluorescence spectra of luminol appeared at around 425 nm [35]. Upon gradual addition of dsDNA, the fluorescence intensity decreased without any change in wavelength and pattern. It proved that dsDNA could also quench the fluorescence of luminol. The quenching efficiency was evaluated by Stern-Volmer method as the insert of Figure 6. It implies there must be a static quenching from dsDNA for fluorescence of luminol. The bimolecular conjunction rate constant (K_A_) in this case equales to 3.5 × 10^3^ L mol^−1^. It could be attributed to the interaction between luminol and the phosphate backbone of DNA. The –NH– group presenting in luminol molecule interacted with the –P=O group in the DNA strands by hydrogen-bond. It is in well agreement with UV-Vis discussion.

Based on above mentioned discussions, the ECL quenching mechanism of DNA for luminol can be proposed as in the following scheme:

**Scheme 1.**
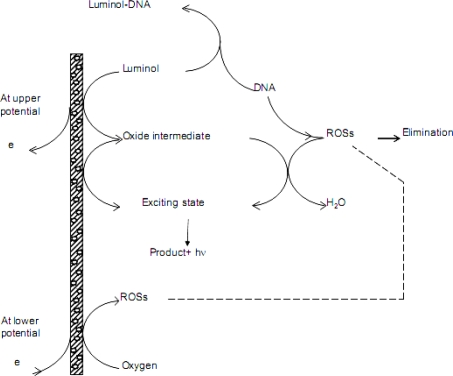
The mechanism of the ECL response of luminol on proposed sensor.

## 4. Conclusions

The dsDNA could effectively quench the ECL of luminol in the pH range of 6.5 to 11. This might be due to the adsorptive interaction between luminol molecules and dsDNA and the elimination of ROSs by dsDNA. In this research, 1.0 × 10^−12^ to 2.0 × 10^−11^ molar of dsDNA on nano-composite pre-functional glassy carbon electrode surface resulted in a remarkable decrease of ECL intensity. It presented the linearity upon dsDNA amount with reciprocal of ECL intensity. A saturated modified electrode caused a 92.8% quenching effect. It could be inferred that sensitive sensing for dsDNA can be expected, so this ECL sensor is expected to have the potential for real applications in molecular biology. The DNA sensor offers excellent stability and reproducibility, and could potentially serve as a powerful tool for the label-free investigation of dsDNA. Finally, the simply designed device might encourage the further development and applications in clinical practice, medicine and basic research.
